# The Immunological Impact of Adenovirus Early Genes on Vaccine-Induced Responses in Mice and Nonhuman Primates

**DOI:** 10.1128/JVI.02253-20

**Published:** 2021-03-10

**Authors:** Kotou Sangare, Iskra Tuero, Mohammad Arif Rahman, Tanya Hoang, Leia K. Miller-Novak, Diego A. Vargas-Inchaustegui, David J. Venzon, Celia LaBranche, David C. Montefiori, Marjorie Robert-Guroff, Michael A. Thomas

**Affiliations:** a Department of Biology, Howard University, Washington, DC, USA; b Section on Immune Biology of Retroviral Infection, Vaccine Branch, National Cancer Institute, National Institutes of Health, Bethesda, Maryland, USA; c Biostatistics and Data Management Section, National Cancer Institute, National Institutes of Health, Bethesda, Maryland, USA; d Duke University Medical Center, Durham, North Carolina, USA; Emory University

**Keywords:** adenovirus, HIV, vaccine, nonhuman primates, early region 1B55K (E1B55K), early region 4 (E4), immune responses, antibody, cytokine genes, cytokine-producing cells, rhFLSC

## Abstract

Adenovirus (Ad) is being explored for use in the prevention and treatment of a variety of infectious diseases and cancers. Ad with a deletion in early region 3 (Δ*E3*) provokes a stronger immune response than Ad with deletions in early regions 1 and E3 (Δ*E1*/Δ*E3*). The ΔE1/ΔE3 Ads are more popular because they can carry a larger transgene and, because of the deleted E1 (E1A and E1B), are perceived safer for clinical use. Ad with a deletion in E1B55K (ΔE1B55K) has been in phase 3 clinical trials for use in cancer therapy in the United States and has been approved for use in head and neck tumor therapy in China, demonstrating that Ads containing E1A are safe for clinical use. We have shown previously that ΔE1B55K Ad, even while promoting lower levels of an inserted transgene, promoted levels of transgene-specific immune responses similar to those of a Δ*E3* Ad. Products of the Ad early region 4 (*E4*) limit the ability of cells to mount an innate immune response. Using this knowledge, we deleted Ad E4 open reading frames 1 (E4orf1) to E4orf4 (E4orf1-4) from the ΔE1B55K Ad. Here, we show that innate cytokine network genes are elevated in Δ*E4* Ad-infected cells beyond that of Δ*E3* Ad-infected cells. Furthermore, in immunized mice, the IgG2a subclass was favored, as was the IgG1 subclass, in immunized nonhuman primates. Thus, Ad E4 impacts immune responses in cells, immunized mice, and immunized nonhuman primates. These Ads may offer advantages that are beneficial for clinical use.

**IMPORTANCE** Adenovirus (Ad) is being explored for use in the prevention and treatment of a variety of infectious diseases and cancers. Here, we provide evidence in cells, mice, and nonhuman primates supporting the notion that Ad early gene products limit specific immune responses. Ad constructed with deletions in early genes and expressing HIV envelope protein was shown to induce greater HIV-specific cellular immune responses and higher-titer antibodies than the parental Ad with the early genes. In addition to eliciting enhanced immunity, the deleted Ad possesses more space for the insertion of additional or larger transgenes needed for targeting other infectious agents or cancers.

## Abstract (precis)

Adenovirus (Ad) is being explored for use in the prevention and treatment of a variety of infectious diseases and cancers. Here, we provide evidence in cells, mice, and nonhuman primates supporting the notion that Ad early gene products limit specific immune responses.

## INTRODUCTION

Adenovirus (Ad) is being developed as a component of vaccine strategies for human immunodeficiency virus (HIV) (1–3), influenza virus (4–6), Mycobacterium tuberculosis (7, 8), and many other infectious diseases (9–14) as well as for cancers (15, 16) because of its ability to prime immune responses (2, 17). Despite this widespread use, the current understanding of the effects of Ad genes on the immune responses induced against the Ad vector and/or the transgene expressed remains somewhat incomplete.

Ad is a linear double-stranded DNA-containing virus. After the genomic DNA is deposited into the nucleus, transcription of the viral genes begins with the immediate early region 1A (E1A). E1A induces the activation of the other early genes (*E1B*, *E2*, *E3*, and *E4*) (18). Even without *E1A*, (*E1*-deleted) Ad vectors are able to induce strong immune responses against Ad itself and products of an inserted transgene (19).

The E1 products transform rodent cells (20, 21). Therefore, for safety reasons, in addition to its ∼5-kb transgene-carrying capacity, *E1*-deleted Ads (Δ*E1* Ads) are the most common form of Ad currently being explored for clinical use (22). However, evidence, including the fact that Ads with a deletion in *E1B55K* (Δ*E1B55K* Ad) have made it to phase 1, 2, and 3 clinical trials in the United States and are used in China for the treatment of head and neck cancer (23), makes the case that E1A-containing Ads are safe for clinical use.

Previously, to expand the use of Δ*E1B55K* Ad, we replaced the Ad *E3* with full-length single-chain HIV_BaL_gp120 linked to rhesus macaque CD4 (rhFLSC) (24–26) and showed that even while producing a smaller amount of the HIV transgene in infected cells, mice immunized with this construct produced levels of cytokine-producing T cells and binding antibodies similar to those produced by mice immunized with the Δ*E3* Ad (24).

Products of the Ad early region 4 (*E4*) are reported to limit the ability of Ad-infected cells to mount an innate immune response (27–29). Based on this knowledge, we hypothesized that deleting the E4 genes presented a way to enhance the immune-priming ability of the Δ*E1B55K* Ad while simultaneously expanding the transgene-carrying capacity from ∼3.8 kb to ∼5.2 kb. For some time, we have used the Ad type 5 (Ad5) host range mutant (Ad5hr) (30), which can replicate in rhesus macaques, as a model for the development of HIV vaccine strategies (31–33). Here, using this Ad5hr, we deleted the DNA sequences for the *E1B55K* and *E4orf1* to *E4orf4* (*E4orf1-4*) genes and replaced the *E3* genes with rhFLSC. For simplicity, we call this construct Δ*E4* Ad. Cells infected with the Δ*E4* Ad expressed higher levels of innate cytokine network genes than cells infected with the Δ*E3* Ad. Mice inoculated with the Δ*E4* Ad exhibited higher levels of HIV-specific IgG2a binding antibodies than the Δ*E3* Ad-immunized mice. What is more, nonhuman primates (NHPs) inoculated with the Δ*E4* Ad displayed higher levels of HIV-specific cytokine-producing mucosal T cells and HIV-specific IgG1 antibodies than Δ*E3* Ad-immunized NHPs. Thus, we created Ad vaccine candidates with an expanded transgene-carrying capacity and show here, in cultured cells, mice, and nonhuman primates, evidence that Ad early genes impact the immune responses generated.

## RESULTS

### Ad early genes modulate levels of viral late proteins and viral progeny production.

**FIG 1:**
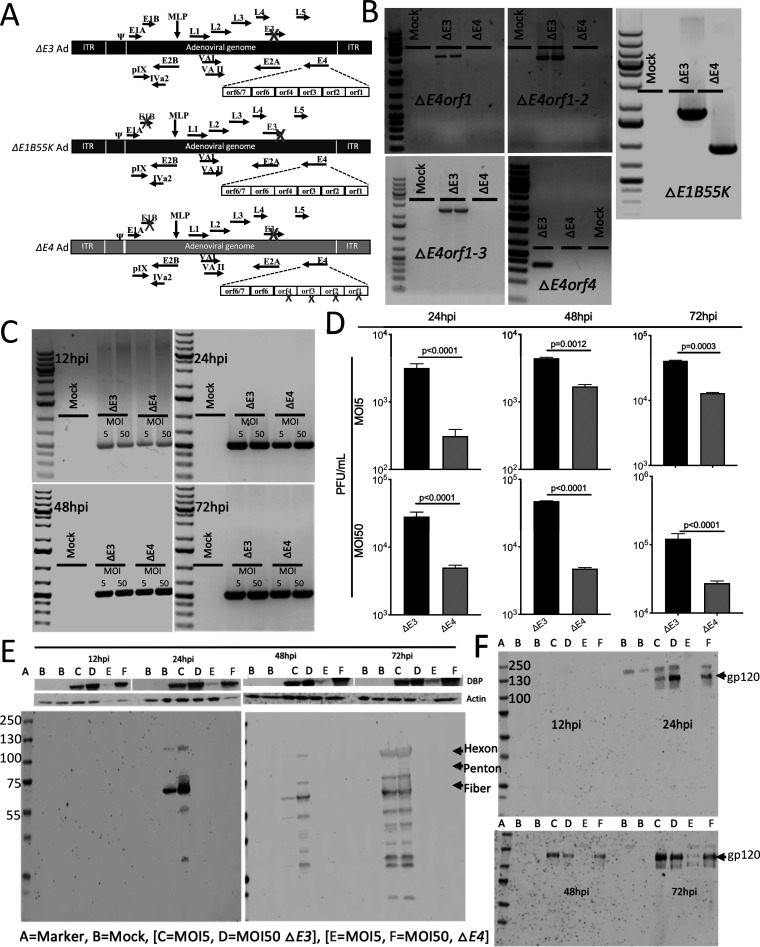
Characterization of Ad5 HIV vaccine candidates in infected human lung epithelial cells. (A) Adenovirus genome, with “X” indicating deleted genes and ITR inverted terminal repeat. (B to F) A549 cells were infected at an MOI of 5 or 50 for 24, 48, and 72 h. (B) Previously described E4orf1, 1-2, 1-3, E4orf4, and E1B55K primers (24, 37) were used in PCR experiments to reveal the presence or absence of the indicated genes. (C) PCR for a segment of the Ad fiber gene was performed. (D) Plaques were counted, and mean values ± standard errors of the means (SEM) are plotted. (E and F) The static levels of Ad DNA binding protein (DBP), actin, and Ad late proteins (hexon, penton, and fiber) (E) and levels of gp120 (F) were assessed by Western blotting. All experiments were repeated at least 3 times. Representative examples are shown in panels B, C, E, and F. *P* values were obtained by ANOVA after Bonferroni correction.

Ads with deletions in *E1B55K* and the different *E4* genes have been previously described (34–36). However, because they lacked a transgene, whether the E4 products play a role in the Ad-induced transgene-specific immune responses has remained somewhat unclear. In Fig. 1A, we provide schematics of the genomes of the Ads used in this study. The Δ*E3* (24, 37, 38) and Δ*E1B55K* (24) Ads were previously described. In Fig. 1, we characterize the Δ*E1B55K*/Δ*E3*/Δ*E4orf1-4* Ad (termed Δ*E4* from this point on) in A549 cells. Previously described primers (24, 37) were used in PCR experiments to reveal the deletion of the Ad *E4orf1*, *E4orf1* to *E4orf2*, *E4orf1* to *E4orf3*, and *E4orf1* to *E4orf4* genes as well as E1B55K (Fig. 1B). At a high multiplicity of infection (MOI), the lack of differences in levels of Ad fiber DNA (Fig. 1C) and RNA (data not shown) in the Δ*E4* Ad-infected cells does not predict the differences in the progeny yields shown in Fig. 1D. Here, compared to the Δ*E3* Ad, the Δ*E4* Ad promotes significantly smaller amounts of progeny (Fig. 1D). No discernible differences in the levels of Ad DNA binding protein (DBP) encoded by Ad early region 2A were observed at a high MOI (Fig. 1E). This was in stark contrast to the diminished levels of Ad late proteins, for example, hexon, penton, and fiber, observed over time. In the Δ*E4* Ad-infected cells, levels of Ad late proteins remained lower than those observed in Δ*E3*-infected cells even at 72 h postinfection (hpi) (Fig. 1E). rhFLSC (data not shown) and gp120 (Fig. 1F) are expressed around the same time as the Ad late proteins, as reported previously for Δ*E1B55K* Ad (24), and were also diminished in Δ*E4* Ad-infected cells compared to Δ*E3* Ad-infected cells. Similar results were observed in HeLa, TC1, and CV1 cells (data not shown). We did not measure any later times. However, from these results, it appears that levels of viral progeny measured in Δ*E4* Ad-infected cells (Fig. 1D) closely parallel the diminished levels of Ad late proteins (Fig. 1E), as previously reported (36, 39).

### Ad early genes modulate levels and types of cytokines produced during infection.

**FIG 2:**
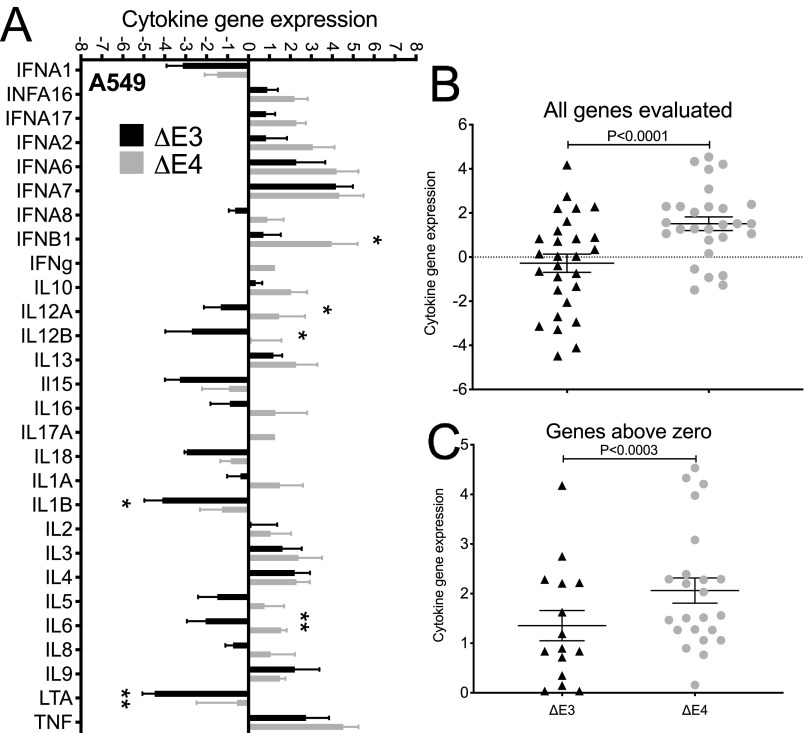
Expression levels of innate cytokine network genes in Ad-infected human epithelial cells. A549 cells were infected at an MOI of 50 with Δ*E3* or Δ*E4* Ad for 48 h. Total RNA was isolated and reverse transcribed to cDNA. The fold change was determined using the 2^−ΔΔ^*^CT^* method. (A) *P* values were obtained using multiple *t* tests. (B and C) The mean values of all the innate cytokines measured from Δ*E4*- and Δ*E3*-infected A549 cells (B) as well as those above zero from both vectors (C) were compared. *P* values were obtained using the Wilcoxon matched-pairs signed-rank test. For each experiment, 3 biological replicates were performed and analyzed. *, *P* < 0.05; **, *P* < 0.01.

Cytokines are an important part of the initial cascade of molecules that communicate the presence of an ongoing infection. The *E1B55K* (24, 40, 41) and *E4* (27–29, 42) gene products are reported to limit the ability of Ad-infected cells to mount an innate immune response. Yet the effects of the deletion of these genes on the levels of innate cytokine genes during Ad infection were never cataloged. A comparison of 28 innate cytokine network genes evaluated in A549 cells revealed significant differences in the abilities of the two vaccine candidates to control the levels of interferon beta (IFN-β), interleukin 12 (IL-12), interleukin 6 (IL-6), interleukin 1 (IL-1), and lymphotoxin alpha (LTA) (Fig. 2A). The bars with values below zero represent genes that are suppressed. Of these, 13 were from Δ*E3* Ad infections, but only 5 were from the Δ*E4* Ad infection (Fig. 2A), for an overall significant difference of a *P* value of <0.0001 (Fig. 2B). The bars with values above zero include the innate cytokine network genes that were induced. Significantly more of these were from cells infected with the Δ*E4* Ad (Fig. 2C) (*P* < 0.0003). From Fig. 2, it is clear that infections with the Δ*E4* Ad allow for the expression of higher numbers as well as in some cases higher levels of innate cytokine network genes than infections with Δ*E3* Ad. These findings suggest that *E1B55K* and *E4* gene products may act to suppress cytokine gene expression in Ad-infected cells.

### Ad early genes modulate levels of transgene-specific antibodies produced in immunized mice.

**FIG 3:**
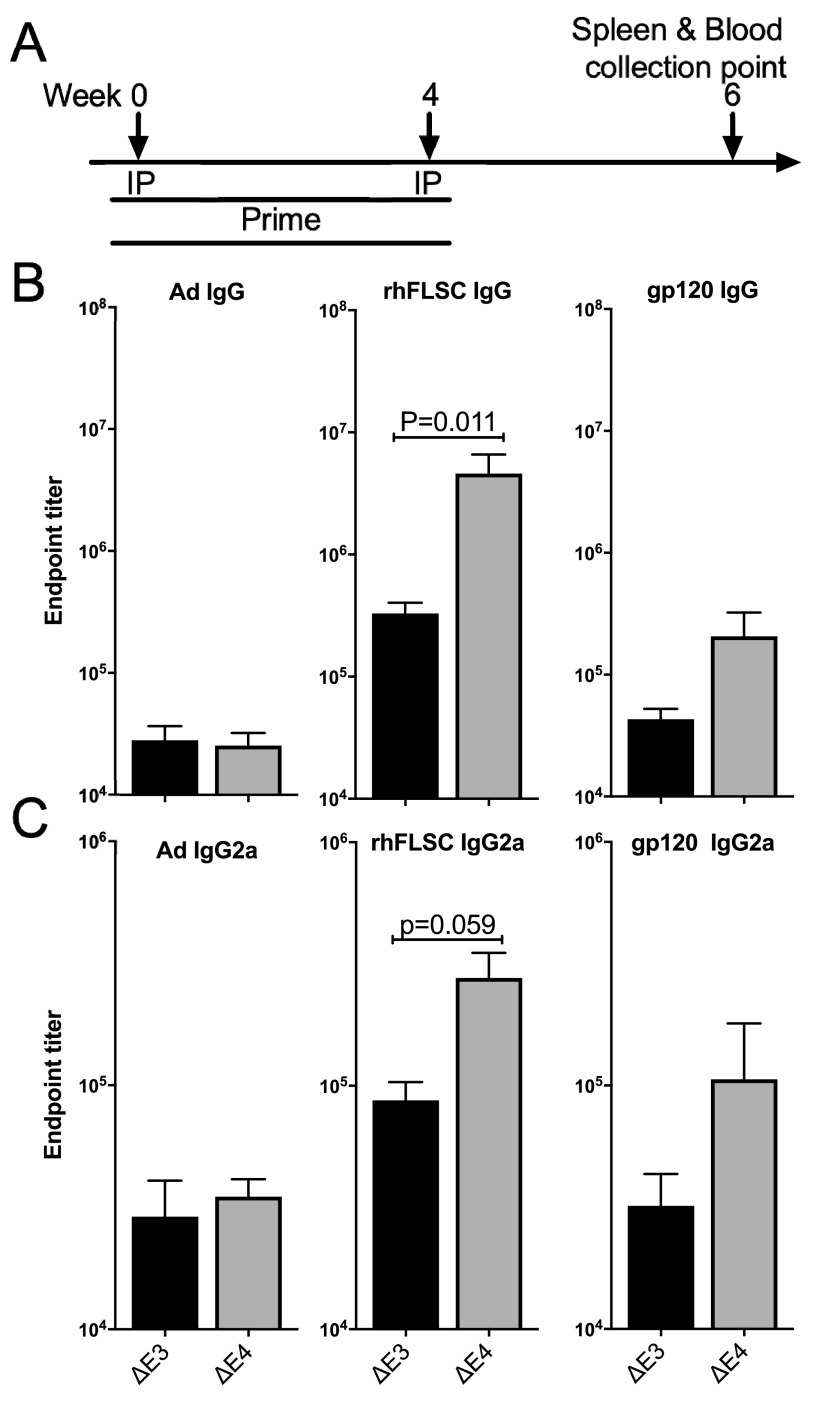
Levels of Env-specific serum binding antibodies in immunized mice. (A) Randomly assigned experimental groups of 5 mice were inoculated intraperitoneally (IP) at weeks 0 and 4 with 5.0 × 10^8^ PFU Δ*E3* or Δ*E4* Ad per mouse. Five naive mice served as controls. Blood was collected at week 6. (B) Titers of Ad-, rhFLSC-, and gp120-specific IgG antibodies were measured by an ELISA. (C) Titers of Ad-, rhFLSC-, and gp120-specific serum IgG2a antibodies were measured by an ELISA. The values for the control mice were below the background and are not shown. Results are presented as means ± SEM. *P* values were obtained using 2-way ANOVA.

Ad5hr vaccine candidates that express rhFLSC promote high levels of HIV-specific antibodies in mice (24, 37) and NHPs (38). The antibodies elicited by the rhFLSC immunogen are capable of neutralizing HIV (25, 38). Thus, an enhancement of the levels of rhFLSC antibodies induced by vaccines may prove important. To determine the effects of deleting Ad *E1B55K* and *E4orf1-4* on antibody induction, mice were inoculated intraperitoneally with either Δ*E3* Ad or the Δ*E4* Ad, as shown in Fig. 3A. The titers of Ad-specific and rhFLSC-specific serum antibodies induced were compared (Fig. 3B). Mice immunized with the Δ*E4* Ad produced levels of Ad-specific IgG antibodies similar to those of mice immunized with Δ*E3* Ad. In contrast, mice inoculated with the Δ*E4* Ad produced a >10-fold increase in IgG antibody titers specific for rhFLSC compared to mice inoculated with Δ*E3* Ad (*P* = 0.011) (Fig. 3B).

The rhFLSC construct is designed to present epitopes that are exposed by the binding of CD4 to the HIV envelope (CD4 induced [CD4i]) (24, 25). These conserved epitopes are important targets of the adaptive humoral immune response and induce broadly cross-reactive antibodies against the HIV-1 envelope glycoprotein (38, 43). However, as the rhesus CD4 component of the rhFLSC immunogen is foreign to mice, it serves as an additional antibody target, leading to high IgG titers in both the ΔE3- and ΔE4-immunized mice compared to the IgG titers against Ad and gp120 itself (Fig. 3B). Nevertheless, the gp120-specific IgG titers retained the same pattern as that seen for the rhFLSC-specific IgG antibodies, with higher titers observed following immunization with the Δ*E4* Ad (Fig. 3B).

In mice, of the five IgG subclasses, IgG2a is reported to clear viral pathogens (44, 45). Therefore, we assessed the levels of Ad- and HIV-specific IgG2a antibodies produced in the immunized mice. Mice immunized with the Δ*E4* Ad promoted high levels of rhFLSC-specific IgG2a approaching significance (*P* = 0.059) compared to Δ*E3* Ad-immunized mice (Fig. 3C). IgG2a antibodies specific for gp120 exhibited a similar pattern. Thus, based on the rhFLSC data, products of the *E4* genes appear to suppress transgene-specific antibody production, including the IgG2a subclass reported to be important for viral clearance in mice (44, 45).

### Ad early genes modulate levels of transgene-specific cytokine-producing cells in the mucosa of immunized NHPs.

**FIG 4:**
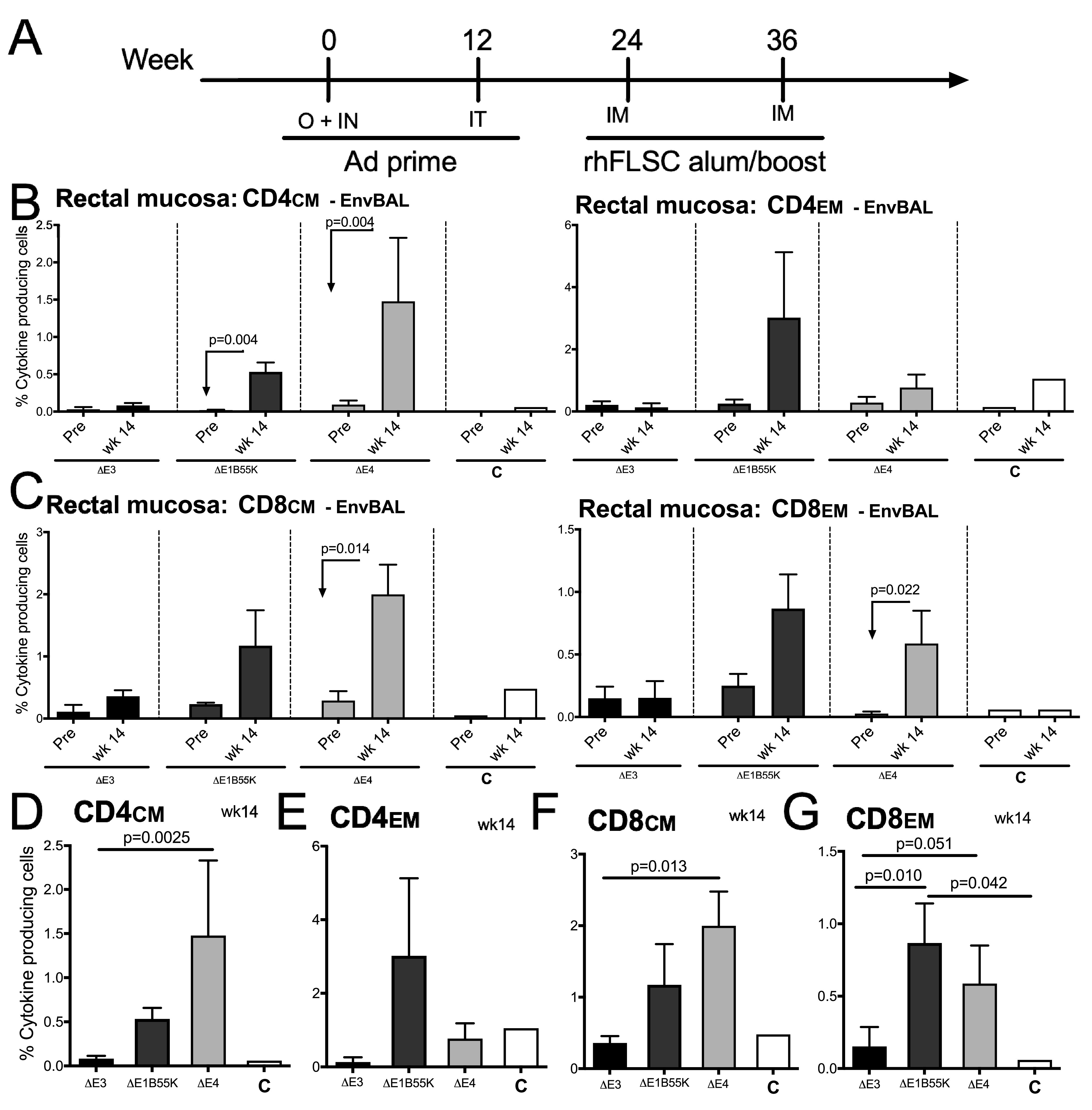
Levels of Env-specific cytokine-producing mucosal memory CD4 and CD8 cells in immunized rhesus macaques. (A) Three macaques per group were immunized at week 0 intranasally (IN) and orally (O) and at week 12 intratracheally (IT) with Δ*E3*, Δ*E1B55K*, or Δ*E4* Ad as described in Materials and Methods. One control macaque, C, received empty Ad5hr. The rhFLSC boost in alum was administered intramuscularly (IM). (B and C) The mean percentages ± SEM of cytokine (TNF-α, IL-2, and IFN-γ)-producing rectal CM and EM CD4 and CD8 T cells prior to immunization (pre) and at week 14 for each immunization group are shown. (D to G) The mean percentages ± SEM of cytokine (TNF-α, IL-2, and IFN-γ)-producing T cells on week 14 are compared across immunization groups.

Vaccine-induced cellular immunity is also important for protective efficacy. We wanted to assess cellular immunity induced by the mucosal Ad immunizations at a mucosal site. In order to obtain sufficient mucosal cells and also because the replication of human Ad is restricted in mice (46), we assessed the levels of HIV_BaL_gp120-specific cytokine-producing T cells in rectal cells of rhesus macaques permissive for Ad5hr replication (30). In this pilot study, groups of 3 rhesus macaques each were primed mucosally at weeks 0 and 12 with either Δ*E3* Ad, a Δ*E1B55K* Ad, or the Δ*E4* Ad and boosted intramuscularly at weeks 24 and 36 with rhFLSC protein in alum adjuvant (Fig. 4A). This mucosal immunization strategy has been shown to prime both systemic and mucosal responses (38, 43). A control macaque received the empty Δ*E3* Ad vector and alum only. Using intracellular cytokine staining (ICS) and flow cytometry, we quantified the percentages of mucosal cytokine-producing central memory (CM) and effector memory (EM) CD4^+^ T cells specific for HIV_BaL_gp120 (Fig. 4B). Two weeks after the second priming (week 14), the levels of cytokine-producing CD4 CM T cells increased in the NHPs that were inoculated with the Δ*E1B55K* and Δ*E4* Ads compared to preimmunization levels (Fig. 4B) (*P* = 0.004 for both). The response patterns of both CM and EM CD8^+^ T cells were also significantly increased at week 14 in macaques immunized with the Δ*E4* Ad (*P* = 0.014 and *P* = 0.022, respectively) (Fig. 4C). At week 14, the levels of CD4^+^ CM T cells producing the three cytokines measured were significantly higher in NHPs immunized with the Δ*E4* Ad than in NHPs immunized with Δ*E3* Ad (Fig. 4D) (*P* = 0.0025). Both CD8^+^ CM (Fig. 4F) (*P* = 0.013) and EM (Fig. 4G) (*P* = 0.051) T cells from macaques immunized with the Δ*E4* Ad expressed higher levels of cytokines than those from Δ*E3* Ad-immunized NHPs. While the numbers of NHPs used were low, these results suggest that products of the Ad *E1B55K* and *E4* genes may act to suppress the levels of mucosal cytokine-producing memory CD4^+^ and CD8^+^ T cells in immunized NHPs.

### Ad early genes modulate levels of transgene-specific antibodies produced in immunized NHPs.

**FIG 5:**
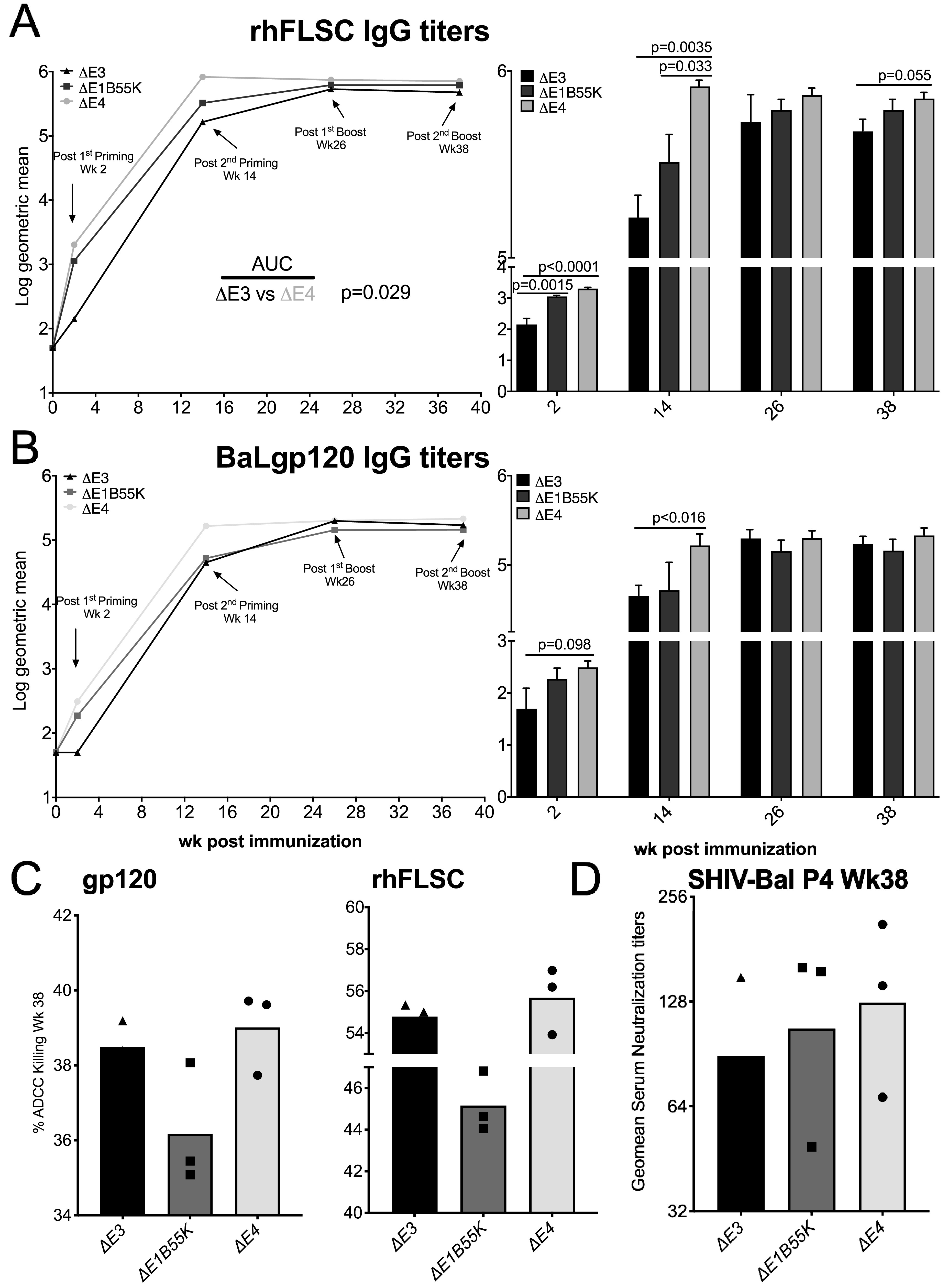
Levels of Env-specific IgG antibody in immunized rhesus macaques. (A and B) Serum levels of rhFLSC (A)- and gp120 (B)-specific IgG antibodies were measured by an ELISA. Results are displayed as log_10_ geometric means. (C) Mean values of week 38 ADCC for gp120- and rhFLSC-coated target cells are shown. (D) Geometric mean values of SHIV_BaL_-P4 week 38 serum neutralization titers are shown. *P* values were obtained using repeated-measures ANOVA and the Wilcoxon-Mann-Whitney test.

To assess the ability of the Ad vaccine candidates to promote a humoral response in NHPs, we determined the titers of rhFLSC- and gp120-specific serum binding antibodies produced over the course of the immunization by an enzyme-linked immunosorbent assay (ELISA). In Fig. 5, consistent with the T-cell data in Fig. 4, macaques immunized with Δ*E1B55K* or the Δ*E4* Ad exhibited significant enhancements (*P* = 0.0015 and *P* < 0.0001, respectively) in rhFLSC-specific antibodies after the first priming inoculation compared to the levels elicited by Δ*E3* Ad (Fig. 5A). Two weeks after the second inoculation, the differences in antibody levels induced by the Δ*E4* Ad compared to Δ*E3* Ad remained significant (*P* = 0.0035) and were also higher than those in the Δ*E1B55K* Ad-immunized macaques (*P* = 0.033). This pattern continued into the boosting phase of the vaccine regimen, where the antibody levels induced by the Δ*E4* Ad remained elevated compared to the Δ*E3* Ad (after the 2nd boost, *P* = 0.055). Throughout the study, the area under the curve (AUC) of rhFLSC-specific antibody induced by the Δ*E4* Ad remained significantly higher (*P* = 0.029) than that induced by Δ*E3* Ad (Fig. 5A).

We measured and compared the levels of HIV_BaL_gp120-specific antibodies induced by each of the vaccine candidates (Fig. 5B). As in the above-described mouse study, the levels of gp120-specific antibodies were lower than those induced against the rhFLSC immunogen. However, levels of gp120-specific IgG induced in Δ*E4* Ad-immunized NHPs remained higher than those observed for Δ*E3* Ad-immunized NHPs (after the 1st priming, *P* = 0.098; after the 2nd priming, *P* < 0.016) (Fig. 5B).

Binding antibodies to variable regions 1 and 2 of the HIV envelope were among the only correlates of protection in the RV144 phase 3 HIV vaccine trial (47), where 31% vaccine efficacy was shown (48). So far, our results suggest that deleting Ad *E1B55K* and the *E4* genes represents a means to enhance transgene-specific antibody responses in immunized NHPs. Among the functions that binding antibodies participate in, antibody-dependent cell-mediated cytotoxicity (ADCC) may have a beneficial impact on HIV infection (49, 50). ADCC occurs when the Fc of the antibody forms a bridge between a target cell (bearing the HIV antigens on its surface) and an effector cell expressing the Fc receptor (51). Ligation to the Fc receptor initiates a cascade of signals that results in the lysis of the target cell. We have previously shown that antibodies induced by the Δ*E3* Ad are capable of promoting cell killing through ADCC (38). Thus, to determine if the E4 genes had any impact on this ability, we performed an ADCC assay, as summarized in Materials and Methods and described previously (52). Here, even though the ADCC values from the Δ*E4* Ad-immunized NHPs were higher than those from the other Ad-immunized NHPs, no significant differences were observed against either gp120 or rhFLSC targets (Fig. 5C).

Some of the binding antibodies produced by the Ad-immunized NHPs may have the ability to neutralize and otherwise inactivate HIV. To assess whether the E4 genes impacted this ability, we performed neutralization assays as described previously (38). As with the ADCC results, the geometric mean neutralizing serum titer from the Δ*E4* Ad-immunized NHPs was slightly higher than that from the other Ad-immunized NHPs but not significantly (Fig. 5D).

### Ad E4 genes modulate IgG1-specific antibodies produced in immunized NHPs.

**FIG 6:**
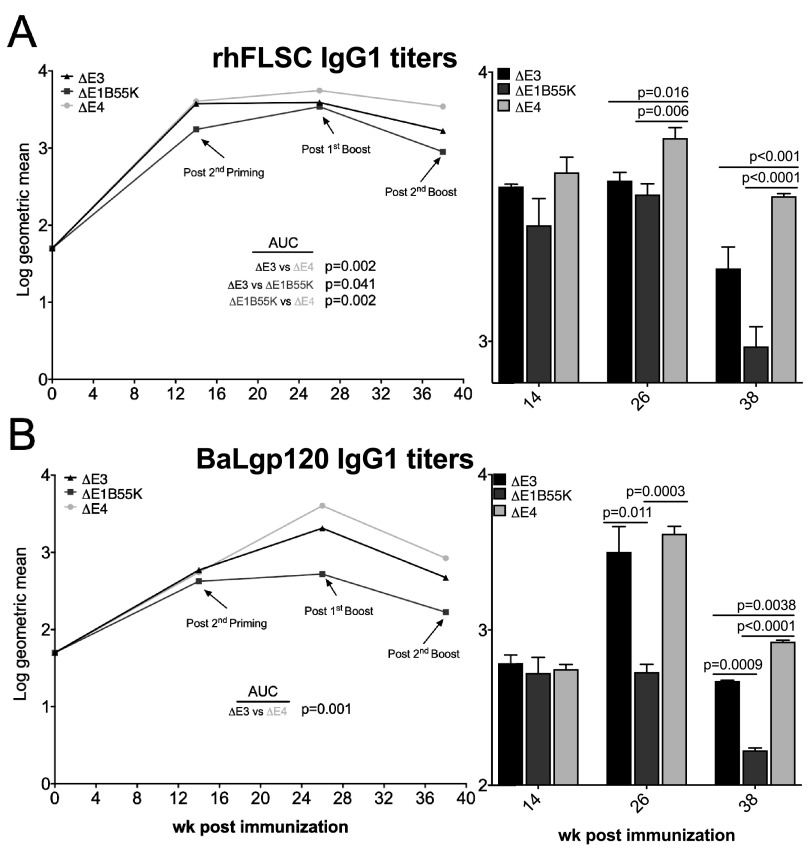
Levels of Env-specific IgG1 antibody in immunized rhesus macaques. Serum levels of rhFLSC (A)- and gp120 (B)-specific IgG1 antibodies were measured by an ELISA. Results are displayed as log_10_ geometric means. *P* values were obtained using repeated-measures ANOVA and the Wilcoxon-Mann-Whitney test.

Because IgG2a, reported to be important for clearing viral pathogens (44, 45), was induced to higher levels by Δ*E4* Ad than by Δ*E3* Ad, the Δ*E4* Ad may have also promoted the equivalent antibody subtype in NHPs. Similar to the IgG2a of mice, the IgG1 subclass antibodies of NHPs exhibit the broadest function (53). In Fig. 6A and B, we show that NHPs inoculated with the Δ*E4* Ad continued to produce high levels of IgG1 against the HIV transgene for weeks longer than Δ*E3* Ad. For example, 2 weeks following the 2nd boost, rhFLSC-specific (Fig. 6A) and BaLgp120-specific (Fig. 6B) IgG1 titers induced by the Δ*E4* Ad were significantly higher than those against both the Δ*E3* Ad (*P* < 0.001 and *P* = 0.0038, respectively) and the Δ*E1B55K* Ad (*P* < 0.0001 for both). Unlike the levels of HIV-specific IgG shown in Fig. 5, in Fig. 6A, the levels of rhFLSC-specific IgG1 elicited in the Δ*E1B55K* Ad-immunized NHPs were consistently lower than those elicited in NHPs inoculated with Δ*E3* Ad (*P* = 0.041). Moreover, levels of rhFLSC-specific IgG1 elicited by the Δ*E3* Ad were lower than those elicited in NHPs inoculated with the Δ*E4* Ad (*P* = 0.002). Analysis of BaLgp120-specific IgG1 antibodies (Fig. 6B) confirmed the induction of higher titers by the Δ*E4* Ad than by the Δ*E3* Ad (*P* = 0.001). Thus, in Ads that contain the same surface proteins, products of the *E4* genes seem to act to suppress subtype-specific antibody responses.

## DISCUSSION

Ad vectors are being developed as part of a vaccine strategy for a variety of different maladies because they evoke strong transgene-focused immune responses. Here, we provide evidence across cells, mice, and nonhuman primates showing that Ad early genes (namely, *E1B55K* and *E4orf1-4*) impact the nature of the induced transgene-specific immune responses.

Our findings follow a previous study in mice that showed that deletion of the Ad *E4* genes blunts host immune responses induced by Δ*E1* Ad (54). One difference between the studies may be that all our vectors contain E1A. E1A-containing Ads are being explored for use in vaccine delivery (5, 6, 55–58) and exhibit potent immunogenicity. In one study, a Δ*E3* Ad-HIV recombinant was shown to induce a higher frequency of HIV Env-specific interferon gamma-secreting lymphocytes and T-cell proliferative responses, higher anti-Env binding and neutralizing antibody titers, and better induction of antibody-dependent cellular cytotoxicity than a Δ*E1*/Δ*E3* Ad (59). The two vectors, with and without *E1*, respectively, contained the same *E4*, suggesting that activities associated with the Ad *E1* (E1A and/or E1B) provoked transgene-specific cellular and adaptive immune responses. Also, in the Δ*E4* Ad, we retained *E4orf6*. E4orf6 has been reported to cooperate with the E1A proteins (60). So E4orf6 may act to enhance the activity of E1A, while E1B55K along with E4orf1-4 act to counter the effects (Fig. 2 to 6).

We have shown that cells infected with Ad with deletions in *E1B55K* produced higher levels of cytokines and displayed higher levels of NK cell-activating markers on their surfaces than cells infected with the Δ*E3* Ad (24). This, as well as the enhanced cellular (Fig. 4) and early antibody (Fig. 5A and B) responses elicited by the Δ*E1B55K* Ad, suggests that E1B55K acts to inhibit Ad-induced immune activation. As we show in Fig. 2, many of the cytokine genes that exhibited elevated expression in Δ*E1B55K* Ad-infected cells (such as interferon [IFN], interleukin 12 [IL-12], tumor necrosis factor alpha [TNF-α], and IL-6) (24) also exhibited enhanced expression during infection with Δ*E4* Ad. If produced by susceptible cells in mice or NHPs, these may mediate innate immune cell attraction and activation (61). IFN-β1 activates Janus kinases, leading to the phosphorylation and activation of signal transducer and activator of transcription 1 (STAT1), STAT2, and STAT3, resulting in the expression of hundreds of IFN-responsive genes (62). IL-12 is a heterodimeric cytokine of 70 kDa comprising two covalently linked subunits, p35 and p40 (IL-12A and IL-12B). IL-12 receptors are located mainly on T cells and NK cells and stimulate the Th1 phenotype. The TNF superfamily is involved in the regulation of multiple biological processes, including cell proliferation, differentiation, and cell death. Like TNF, IL-6 is produced by many cell types and attracts innate immune cells. Similar to IL-12, IL-6 is a class I cytokine that uses gp130 in its receptor complex (63, 64) and plays a critical role in immune responses (65, 66).

In a previous publication (24), we compared the immune responses engendered by Δ*E3* Ad to those induced by a Δ*E1B55K* Ad. In that study, the induced HIV-specific immune responses elicited by both Ads were equally high and indistinguishable (24). Here, in Fig. 3, however, the results are very different, showing that the Δ*E4* vaccine candidate promoted elevated levels of binding antibodies that were distinguishable from those induced by the Δ*E3* vaccine candidate. Binding antibodies are among the only correlates of vaccine-induced HIV protection shown in the phase 3 RV144 HIV vaccine trial (47). In a previous study, we noted that sera from mice vaccinated with Ad with deletions of E4orf1-4 differentially recognize Ad antigens and the rhFLSC transgene 39. Here, we show that in the context of the E1B55K deletion, further deletion of E4orf1-4 results in an Ad that preferentially stimulates IgG2a in mice (39) (Fig. 3) and IgG1 in NHPs (Fig. 6). Our assessments of ADCC and neutralizing antibody activity in sera of macaques immunized with the various Ad constructs did not reveal significant differences. However, in view of the differences seen among the groups in IgG1 binding titers, it would have been informative to have isolated the IgG1 induced for evaluation of functionality, including other antibody functions such as antibody-dependent cell-mediated virus inhibition (67), antibody-dependent phagocytic activity (68), or inhibition of transcytosis (69). This remains for future studies. Nevertheless, the data that we provide demonstrate that Ad early genes can impact the levels and quality of the induced immune responses.

One of the greatest advantages of the Δ*E1*/Δ*E3* Ad has been its transgene-carrying capacity of approximately 5 kb. This allows the Δ*E1*/Δ*E3* Ad to be used in instances where large transgenes are to be expressed. The deletion of *E1B55K* (∼0.89 kb), *E3* (∼3.0 kb), and *E4orf1-4* (∼1.2 kb) erased the transgene size advantage of the Δ*E1*/Δ*E3* Ad. Moreover, since the Δ*E3* Ad was shown to induce a more robust transgene-specific immune response than the Δ*E1*/Δ*E3* Ad, and as we show here that in many cases, the transgene-specific immune responses elicited by the Δ*E4* Ad eclipse those induced by Δ*E3* Ad, we believe that the Δ*E4* vector may offer immunological advantages not provided by Ad vectors currently being explored in the clinic for use in vaccine delivery.

Finally, the Ad used here stimulated both T-cell and antibody responses. Both are considered essential for protection from HIV. Our pilot study in rhesus macaques did not include enough animals for evaluation of protective efficacy. Nevertheless, our results represent the first demonstration across cells, mice, and nonhuman primates of a role for Ad early gene products encoded by *E1B55K* and *E4orf1-4* in modulating the quality and magnitude of transgene-specific immune responses.

## MATERIALS AND METHODS

### Ethics statement.

All experiments were approved by the Institutional Biosafety Committee at both Howard University and the National Cancer Institute (NCI). All animal experiments were approved by the NCI Animal Care and Use Committee prior to study initiation under protocols VB-016 and VB-017. The NCI Animal Facility is accredited by the Association for Assessment and Accreditation of Laboratory Animal Care International. The standard practices closely follow recommendations made in the *Guide for the Care and Use of Laboratory Animals* of the U.S. National Institutes of Health (70).

### Immunogens.

The rhFLSC transgene consists of full-length single-chain HIV_BaL_gp120, a flexible linker, and the D1 and D2 domains of rhesus macaque CD4 (25).

### Antibodies.

Anti-actin (Sigma); anti-HIV-1 gp120 (Meridian Life Sciences); anti-human CD4 (hCD4) (R&D Systems); anti-Ad type 5 (Abcam); anti-DBP (gift from David Ornelles); horseradish peroxidase (HRP)-conjugated secondary antibodies, including anti-mouse IgG, anti-human IgG, anti-rabbit IgG, or anti-goat IgG (Invitrogen), as dictated by the primary isotype; mouse anti-rhesus IgG2 (3C10) and mouse anti-rhesus IgG1 (7h11) (AIDS Reagent Resource); anti-CD4 HRP-conjugated anti-mouse total IgG, anti-IgG2a, and CD28-phycoerythrin (PE)-Cy7 (eBioscience); CD3-Alexa Fluor 700, interferon gamma (IFN-γ)-fluorescein isothiocyanate (FITC), interleukin-2 (IL-2)–allophycocyanin (APC), CD4-PE, and IFN-γ–FITC CD95-PE-Cy5 (BD Biosciences); and tumor necrosis factor alpha (TNF-α)-brilliant violet 785 and CD8a-APC/Cy7 (BioLegend) were used.

### Viruses.

The *dl*1520 Ad contains an 827-bp deletion in the region encoding the 55-kDa protein (71). The Δ*E3* virus, previously termed MAd5rhFLSC, was described previously (37). The Δ*E1B55K*/Δ*E3*rhFLSC Ad (Δ*E1B55K* Ad) and Δ*E1B55K*/Δ*E3*/Δ*E4orf1-4*rhFLSC Ad (Δ*E4* Ad) were created by recombining SpeI-digested *dl*1520 DNA with the BamHI-digested pBRAd5hrΔ*E3* (TPL-rhFLSC-pA) plasmid in which we deleted the coding regions for E4orf1-4. The resulting viral DNAs were screened by PCR for the presence or absence of the *E1B55K* and *E4orf1-4* genes (Fig. 1B). All the viruses were amplified and the concentrations were determined at ViraQuest, Inc. (Iowa), using plaque assays.

### Cells.

We used the human cervical carcinoma-derived HeLa cell line (ATCC CCL-2) because most of what is known about Ad is in the context of this cell line. We used the human lung A549 cell line (ATCC CCL-185) as Ad is known to target the upper respiratory airway. We used the murine lung epithelial cell line TC1 (gift from Masaki Terabe, NCI) to simulate infection of mouse cells by Ad5. We used the green monkey kidney-derived CV-1 cell line (ATCC CCL-70) to simulate infection of nonhuman primate cells by Ad5. We used Ad-transformed human embryonic kidney-derived 293 cells (ATCC CRL-1573) as they support the growth of *E1*-deleted viruses. All the cells were maintained as previously described (37).

### Mice.

Six- to eight-week-old female BALB/c mice were housed and maintained in a pathogen-free environment according to the standards of the American Association for Accreditation of Laboratory Animal Care at the National Institutes of Health (NIH) (Bethesda, MD). The protocol was reviewed and approved by the Animal Care and Use Committee prior to implementation (protocol VB-016). Mice were randomly assigned to 5 experimental groups and inoculated intraperitoneally at weeks 0 and 4 with 5.0 × 10^8^ cell-determined PFU of Δ*E3* Ad or Δ*E4* Ad per mouse. Five naive mice served as controls. Spleens and blood were collected at week 6 and treated as previously described (37).

### NHPs.

Ten male rhesus macaques (Macaca mulatta), approximately 2.5 years old and weighing between 3.5 and 4.4 kg at the time of study initiation, were housed and maintained at the NCI Animal Facility, NIH (Bethesda, MD), according to the standards of the American Association for Accreditation of Laboratory Animal Care. All were negative for simian immunodeficiency virus (SIV), simian retrovirus type D, and simian T-cell lymphotropic virus (STLV). The protocol was reviewed and approved by the Animal Care and Use Committee prior to implementation (protocol VB-017). Three macaques per group were immunized at week 0 intranasally and orally and at week 12 intratracheally with Δ*E3* Ad, Δ*E1B55K* Ad, or Δ*E4* Ad at a dose of 5 × 10^8^ cell-determined PFU/recombinant/route. The macaques were boosted intramuscularly at two sites with recombinant rhFLSC protein (Profectus BioSciences, Inc.), with a total of 100 μg/macaque, in alum adjuvant (aluminum hydroxide gel) at weeks 24 and 36. One control macaque (C) received an empty Ad5hr vector (5 × 10^8^ cell-determined PFU/macaque) and adjuvant alone.

### Sample collection.

Peripheral blood mononuclear cells (PBMCs) obtained throughout the immunization course were purified from whole blood by Ficoll gradient centrifugation and used immediately for intracellular cytokine staining assays or viably frozen for later use. The rectal biopsy specimens were processed and the lymphocytes were isolated using a Percoll gradient of 35% and 65% layered solutions. Serum samples were collected, aliquoted, and stored at −70°C until use as described previously (72).

### Intracellular cytokines.

Freshly isolated PBMCs (2 × 10^6^) were stimulated with pools of HIV_BaL_gp120 peptides (Advanced BioScience Laboratories, Inc., Rockville, MD) and stained as described previously (73) except that fluorochromes for CD4 and IFN-γ antibodies were changed to CD4-FITC and IFN-γ–PE. A singlet, followed by live/dead and then lymphocytic gates, was first applied. CD3^+^ T cells were divided into CD4^+^ and CD8^+^ populations, and each population was further subdivided into CD28^+^ CD95^+^ central memory (CM) and CD28^−^ CD95^+^ effector memory (EM) cells. The percentage of cytokine-secreting (IFN-γ, TNF-α, and IL-2) cells in each memory cell subset was then determined following subtraction of the values obtained with nonstimulated samples, and the values were summed. Data were analyzed using FlowJo software (TreeStar, Inc.).

### Antibody analyses.

Antibody binding titers were assayed by an enzyme-linked immunosorbent assay (ELISA) as described previously (37). The plates were read at 450 nm within 30 min on a BioTek ELISA reader (ELx803) and analyzed with Gen5 3.02 software. The endpoint titer was defined as the reciprocal of the serum dilution at which the optical density (OD) of the test serum was twice that of the background OD of the plate.

ADCC activity was assessed as previously described, using EGFP-CEMNKr-CCR5-SNAP cells that constitutively express green fluorescent protein (GFP) as targets (52). Briefly, 1 million target cells were incubated with 50 μg of BaLgp120 or rhFLSC recombinant protein for 2 h at 37°C, washed, and labeled with Snap-Surface Alexa Fluor 647 (catalog number S9136S; New England BioLabs, Ipswich, MA), as recommended by the manufacturer, for 30 min at room temperature (RT). Heat-inactivated plasma samples were serially diluted (7 10-fold dilutions starting at 1:10), and 100 μl was added to a 96-well V-bottom plate (Millipore Sigma). Following this, 5,000 gp120- or rhFLSC-coated target cells (50 μl) and 250,000 human PBMCs (50 μl) as effectors were added to each well to give an effector/target cell (E/T) ratio of 50:1. The plate was incubated at 37°C for 2 h, followed by two phosphate-buffered saline (PBS) washes. The cells were resuspended in 200 μl of a 2% PBS–paraformaldehyde solution and acquired on an LSRII instrument equipped with a high-throughput system (BD Biosciences, San Jose, CA). Specific killing was measured by the loss of GFP from the Alexa Fluor 647-positive (Alexa Fluor 647^+^) target cells. Target and effector cells cultured in the presence of medium were used as negative controls. The ADCC endpoint titer (data not shown) is defined as the reciprocal dilution at which the percent ADCC killing was greater than the mean percent killing of the negative-control wells containing medium and target and effector cells, plus 3 standard deviations.

Neutralizing antibody titers against simian-human immunodeficiency virus SHIV_BaL_-P4 were assayed in TZM-Bl cells as described previously (74), using murine leukemia virus (MLV)-pseudotyped virus as a negative control for non-HIV-specific inhibition of the signal. Titers were defined as the reciprocal serum dilution at which there was a 50% reduction in relative luminescence units compared to virus control wells.

### Western blotting.

Gel electrophoresis and Western blotting were performed as described previously (37).

### DNA extraction and PCR.

DNA was extracted using the Qiagen DNA blood minikit. The purity and quantity of all extracted DNA were determined with a NanoDrop spectrophotometer (Thermo Fisher). The PCR assays were performed as described previously (37), with minor modifications. Briefly, test sample DNA was added to the Dream *Taq* green master mix (Thermo Fisher). Reverse and forward primers for Ad fiber and PCR-quality water (Thermo Fisher) were added to reach a final volume of 25 μl. A negative control (no template DNA) was included in each run. PCR amplification was performed using a DNA thermocycler (Life Technologies). The PCR product was loaded on a 1% agarose gel, stained, and photographed with a Li-Cor imaging system. The molecular weight of the PCR products was determined by comparison to a 1-kb DNA ladder (Thermo Fisher).

### Real-time quantitative PCR.

Cells were not infected (mock) or infected with Δ*E3* Ad or the Δ*E4* Ad at an MOI of 50 PFU/cell for 48 h. Total RNA was isolated with RNAzol reverse transcriptase (RT) (Sigma). The RNA concentrations were determined using a NanoDrop spectrophotometer (Thermo Fisher). The RNA was reverse transcribed to cDNA with a QuantiTect reverse transcription kit (Qiagen). Briefly, samples were treated with 10 U DNase (Pharmacia) for 2 min at 42°C, followed by 30 min of cDNA synthesis and inactivation of the Quantiscript reverse transcriptase.

Gene expression was assayed using a TaqMan array human cytokine network 96-well Fast plate (Thermo Fisher). The TaqMan array plate contains 28 assays for genes associated with pro- and anti-inflammatory cytokines and 4 assays for candidate endogenous control genes. Quantitative real-time PCR amplification was performed using QuantStudio 6 (Applied Biosystems). The PCR program used consisted of sample holding at 50°C for 2 min and then 95°C for 10 min, followed by 40 cycles, each consisting of 95°C for 15 s, 56°C for 15 s, and 62°C for 30 s. All assay mixtures were plated in triplicate. The highest cycle number, 40, was used for all undetermined values. The ΔΔ*C_T_* fold number for each experiment was determined and converted into log_2_.

### Statistical analysis.

Differences between groups or between times within groups were assessed using one-way, two-way, and repeated-measures analyses of variance (ANOVAs) and multiple *t* tests, combining panels with common outcomes for appropriate degrees of freedom. Logarithmic transformation of titers and arcsine transformation of percent data were applied before analysis to reduce skewness in raw data. Corrections for multiple comparisons were made where noted. Wilcoxon-Mann-Whitney and Wilcoxon signed-rank tests were used for selected comparisons of groups of adequate size. Analyses were performed using Prism (GraphPad Software, Inc.) and SAS/STAT software version 9.4 (SAS Institute, Inc.).

## References

[1] Shiver JW, Emini EA. 2004. Recent advances in the development of HIV-1 vaccines using replication-incompetent adenovirus vectors. Annu Rev Med 55:355–372. doi:10.1146/annurev.med.55.091902.104344. 14746526

[2] Barouch DH, Liu J, Li H, Maxfield LF, Abbink P, Lynch DM, Iampietro MJ, SanMiguel A, Seaman MS, Ferrari G, Forthal DN, Ourmanov I, Hirsch VM, Carville A, Mansfield KG, Stablein D, Pau MG, Schuitemaker H, Sadoff JC, Billings EA, Rao M, Robb ML, Kim JH, Marovich MA, Goudsmit J, Michael NL. 2012. Vaccine protection against acquisition of neutralization-resistant SIV challenges in rhesus monkeys. Nature 482:89–93. doi:10.1038/nature10766. 22217938 PMC3271177

[3] Abbink P, Maxfield LF, Ng’ang’a D, Borducchi EN, Iampietro MJ, Bricault CA, Teigler JE, Blackmore S, Parenteau L, Wagh K, Handley SA, Zhao G, Virgin HW, Korber B, Barouch DH. 2015. Construction and evaluation of novel rhesus monkey adenovirus vaccine vectors. J Virol 89:1512–1522. doi:10.1128/JVI.02950-14. 25410856 PMC4300752

[4] Xiang K, Ying G, Yan Z, Shanshan Y, Lei Z, Hongjun L, Maosheng S. 2015. Progress on adenovirus-vectored universal influenza vaccines. Hum Vaccin Immunother 11:1209–1222. doi:10.1080/21645515.2015.1016674. 25876176 PMC4514376

[5] Gurwith M, Lock M, Taylor EM, Ishioka G, Alexander J, Mayall T, Ervin JE, Greenberg RN, Strout C, Treanor JJ, Webby R, Wright PF. 2013. Safety and immunogenicity of an oral, replicating adenovirus serotype 4 vector vaccine for H5N1 influenza: a randomised, double-blind, placebo-controlled, phase 1 study. Lancet Infect Dis 13:238–250. doi:10.1016/S1473-3099(12)70345-6. 23369412 PMC3576519

[6] Weaver EA. 2014. Vaccines within vaccines: the use of adenovirus types 4 and 7 as influenza vaccine vectors. Hum Vaccin Immunother 10:544–556. doi:10.4161/hv.27238. 24280656 PMC4130277

[7] Smaill F, Jeyanathan M, Smieja M, Medina MF, Thanthrige-Don N, Zganiacz A, Yin C, Heriazon A, Damjanovic D, Puri L, Hamid J, Xie F, Foley R, Bramson J, Gauldie J, Xing Z. 2013. A human type 5 adenovirus-based tuberculosis vaccine induces robust T cell responses in humans despite preexisting anti-adenovirus immunity. Sci Transl Med 5:205ra134. doi:10.1126/scitranslmed.3006843. 24089406

[8] Li W, Li M, Deng G, Zhao L, Liu X, Wang Y. 2015. Prime-boost vaccination with bacillus Calmette Guerin and a recombinant adenovirus co-expressing CFP10, ESAT6, Ag85A and Ag85B of Mycobacterium tuberculosis induces robust antigen-specific immune responses in mice. Mol Med Rep 12:3073–3080. doi:10.3892/mmr.2015.3770. 25962477

[9] Ledgerwood JE, Costner P, Desai N, Holman L, Enama ME, Yamshchikov G, Mulangu S, Hu Z, Andrews CA, Sheets RA, Koup RA, Roederer M, Bailer R, Mascola JR, Pau MG, Sullivan NJ, Goudsmit J, Nabel GJ, Graham BS, VRC 205 Study Team. 2010. A replication defective recombinant Ad5 vaccine expressing Ebola virus GP is safe and immunogenic in healthy adults. Vaccine 29:304–313. doi:10.1016/j.vaccine.2010.10.037. 21034824

[10] Wong G, Richardson JS, Pillet S, Racine T, Patel A, Soule G, Ennis J, Turner J, Qiu X, Kobinger GP. 2015. Adenovirus-vectored vaccine provides postexposure protection to Ebola virus-infected nonhuman primates. J Infect Dis 212(Suppl 2):S379–S383. doi:10.1093/infdis/jiv102. 25957963 PMC4564535

[11] Geisbert TW, Bailey M, Hensley L, Asiedu C, Geisbert J, Stanley D, Honko A, Johnson J, Mulangu S, Pau MG, Custers J, Vellinga J, Hendriks J, Jahrling P, Roederer M, Goudsmit J, Koup R, Sullivan NJ. 2011. Recombinant adenovirus serotype 26 (Ad26) and Ad35 vaccine vectors bypass immunity to Ad5 and protect nonhuman primates against ebolavirus challenge. J Virol 85:4222–4233. doi:10.1128/JVI.02407-10. 21325402 PMC3126236

[12] Gomi R, Sharma A, Wu W, Sung B, Worgall S. 2017. Post-exposure immunization by capsid-modified AdC7 vector expressing Pseudomonas aeruginosa OprF clears P. aeruginosa respiratory infection. Vaccine 35:7174–7180. doi:10.1016/j.vaccine.2017.10.078. 29126807 PMC5752147

[13] Wang C, Dulal P, Zhou X, Xiang Z, Goharriz H, Banyard A, Green N, Brunner L, Ventura R, Collin N, Draper SJ, Hill AVS, Ashfield R, Fooks AR, Ertl HC, Douglas AD. 2018. A simian-adenovirus-vectored rabies vaccine suitable for thermostabilisation and clinical development for low-cost single-dose pre-exposure prophylaxis. PLoS Negl Trop Dis 12:e0006870. doi:10.1371/journal.pntd.0006870. 30372438 PMC6224154

[14] Xiang ZQ, Greenberg L, Ertl HC, Rupprecht CE. 2014. Protection of non-human primates against rabies with an adenovirus recombinant vaccine. Virology 450–451:243–249. doi:10.1016/j.virol.2013.12.029. PMC4038128 24503087

[15] Zhang C, Zhou D. 2016. Adenoviral vector-based strategies against infectious disease and cancer. Hum Vaccin Immunother 12:2064–2074. doi:10.1080/21645515.2016.1165908. 27105067 PMC4994731

[16] Fougeroux C, Holst PJ. 2017. Future prospects for the development of cost-effective adenovirus vaccines. Int J Mol Sci 18:686. doi:10.3390/ijms18040686. PMC5412272 28420073

[17] Frahm N, DeCamp AC, Friedrich DP, Carter DK, Defawe OD, Kublin JG, Casimiro DR, Duerr A, Robertson MN, Buchbinder SP, Huang Y, Spies GA, De Rosa SC, McElrath MJ. 2012. Human adenovirus-specific T cells modulate HIV-specific T cell responses to an Ad5-vectored HIV-1 vaccine. J Clin Invest 122:359–367. doi:10.1172/JCI60202. 22201684 PMC3248307

[18] Shenk T, Flint J. 1991. Transcriptional and transforming activities of the adenovirus E1A proteins. Adv Cancer Res 57:47–85. doi:10.1016/s0065-230x(08)60995-1. 1835254

[19] Wilson JM. 1996. Adenoviruses as gene-delivery vehicles. N Engl J Med 334:1185–1187. doi:10.1056/NEJM199605023341809. 8602187

[20] Branton PE, Bayley ST, Graham FL. 1985. Transformation by human adenoviruses. Biochim Biophys Acta 780:67–94. doi:10.1016/0304-419x(84)90007-6. 3886009

[21] van Ormondt H, Maat J, van Beveren CP. 1980. The nucleotide sequence of the transforming early region E1 of adenovirus type 5 DNA. Gene 11:299–309. doi:10.1016/0378-1119(80)90070-0. 6260576

[22] Wold WS, Toth K. 2013. Adenovirus vectors for gene therapy, vaccination and cancer gene therapy. Curr Gene Ther 13:421–433. doi:10.2174/1566523213666131125095046. 24279313 PMC4507798

[23] Garber K. 2006. China approves world’s first oncolytic virus therapy for cancer treatment. J Natl Cancer Inst 98:298–300. doi:10.1093/jnci/djj111. 16507823

[24] Thomas MA, Nyanhete T, Tuero I, Venzon D, Robert-Guroff M. 2016. Beyond oncolytics: E1B55K-deleted adenovirus as a vaccine delivery vector. PLoS One 11:e0158505. doi:10.1371/journal.pone.0158505. 27391605 PMC4938603

[25] Fouts TR, Tuskan R, Godfrey K, Reitz M, Hone D, Lewis GK, DeVico AL. 2000. Expression and characterization of a single-chain polypeptide analogue of the human immunodeficiency virus type 1 gp120-CD4 receptor complex. J Virol 74:11427–11436. doi:10.1128/jvi.74.24.11427-11436.2000. 11090138 PMC112421

[26] DeVico A, Fouts T, Lewis GK, Gallo RC, Godfrey K, Charurat M, Harris I, Galmin L, Pal R. 2007. Antibodies to CD4-induced sites in HIV gp120 correlate with the control of SHIV challenge in macaques vaccinated with subunit immunogens. Proc Natl Acad Sci U S A 104:17477–17482. doi:10.1073/pnas.0707399104. 17956985 PMC2077281

[27] Carvalho T, Seeler JS, Ohman K, Jordan P, Pettersson U, Akusjarvi G, Carmo-Fonseca M, Dejean A. 1995. Targeting of adenovirus E1A and E4-ORF3 proteins to nuclear matrix-associated PML bodies. J Cell Biol 131:45–56. doi:10.1083/jcb.131.1.45. 7559785 PMC2120608

[28] Hoppe A, Beech SJ, Dimmock J, Leppard KN. 2006. Interaction of the adenovirus type 5 E4 Orf3 protein with promyelocytic leukemia protein isoform II is required for ND10 disruption. J Virol 80:3042–3049. doi:10.1128/JVI.80.6.3042-3049.2006. 16501113 PMC1395473

[29] Ullman AJ, Reich NC, Hearing P. 2007. Adenovirus E4 ORF3 protein inhibits the interferon-mediated antiviral response. J Virol 81:4744–4752. doi:10.1128/JVI.02385-06. 17301128 PMC1900183

[30] Klessig DF, Grodzicker T. 1979. Mutations that allow human Ad2 and Ad5 to express late genes in monkey cells map in the viral gene encoding the 72K DNA binding protein. Cell 17:957–966. doi:10.1016/0092-8674(79)90335-0. 114304

[31] Robert-Guroff M, Kaur H, Patterson LJ, Leno M, Conley AJ, McKenna PM, Markham PD, Richardson E, Aldrich K, Arora K, Murty L, Carter L, Zolla-Pazner S, Sinangil F. 1998. Vaccine protection against a heterologous, non-syncytium-inducing, primary human immunodeficiency virus. J Virol 72:10275–10280. doi:10.1128/JVI.72.12.10275-10280.1998. 9811775 PMC110613

[32] Patterson LJ, Malkevitch N, Venzon D, Pinczewski J, Gomez-Roman VR, Wang L, Kalyanaraman VS, Markham PD, Robey FA, Robert-Guroff M. 2004. Protection against mucosal simian immunodeficiency virus SIV_mac251_ challenge by using replicating adenovirus-SIV multigene vaccine priming and subunit boosting. J Virol 78:2212–2221. doi:10.1128/jvi.78.5.2212-2221.2004. 14963117 PMC369221

[33] Malkevitch NV, Patterson LJ, Aldrich MK, Wu Y, Venzon D, Florese RH, Kalyanaraman VS, Pal R, Lee EM, Zhao J, Cristillo A, Robert-Guroff M. 2006. Durable protection of rhesus macaques immunized with a replicating adenovirus-SIV multigene prime/protein boost vaccine regimen against a second SIVmac251 rectal challenge: role of SIV-specific CD8+ T cell responses. Virology 353:83–98. doi:10.1016/j.virol.2006.05.012. 16814356

[34] Huang MM, Hearing P. 1989. Adenovirus early region 4 encodes two gene products with redundant effects in lytic infection. J Virol 63:2605–2615. doi:10.1128/JVI.63.6.2605-2615.1989. 2724411 PMC250738

[35] Bridge E, Ketner G. 1990. Interaction of adenoviral E4 and E1b products in late gene expression. Virology 174:345–353. doi:10.1016/0042-6822(90)90088-9. 2137659

[36] Thomas MA, Broughton RS, Goodrum FD, Ornelles DA. 2009. E4orf1 limits the oncolytic potential of the E1B-55K deletion mutant adenovirus. J Virol 83:2406–2416. doi:10.1128/JVI.01972-08. 19129452 PMC2648266

[37] Thomas MA, Song R, Demberg T, Vargas-Inchaustegui DA, Venzon D, Robert-Guroff M. 2013. Effects of the deletion of early region 4 (E4) open reading frame 1 (orf1), orf1-2, orf1-3 and orf1-4 on virus-host cell interaction, transgene expression, and immunogenicity of replicating adenovirus HIV vaccine vectors. PLoS One 8:e76344. doi:10.1371/journal.pone.0076344. 24143187 PMC3797075

[38] Thomas MA, Tuero I, Demberg T, Vargas-Inchaustegui DA, Musich T, Xiao P, Venzon D, LaBranche C, Montefiori DC, DiPasquale J, Reed SG, DeVico A, Fouts T, Lewis GK, Gallo RC, Robert-Guroff M. 2014. HIV-1 CD4-induced (CD4i) gp120 epitope vaccines promote B and T-cell responses that contribute to reduced viral loads in rhesus macaques. Virology 471–473:81–92. doi:10.1016/j.virol.2014.10.001. PMC4312258 25461534

[39] Flint SJ, Gonzalez RA. 2003. Regulation of mRNA production by the adenoviral E1B 55-kDa and E4 Orf6 proteins. Curr Top Microbiol Immunol 272:287–330. doi:10.1007/978-3-662-05597-7_10. 12747554

[40] Miller DL, Rickards B, Mashiba M, Huang W, Flint SJ. 2009. The adenoviral E1B 55-kilodalton protein controls expression of immune response genes but not p53-dependent transcription. J Virol 83:3591–3603. doi:10.1128/JVI.02269-08. 19211769 PMC2663238

[41] Chahal JS, Gallagher C, DeHart CJ, Flint SJ. 2013. The repression domain of the E1B 55-kilodalton protein participates in countering interferon-induced inhibition of adenovirus replication. J Virol 87:4432–4444. doi:10.1128/JVI.03387-12. 23388716 PMC3624377

[42] Hendrickx R, Stichling N, Koelen J, Kuryk L, Lipiec A, Greber UF. 2014. Innate immunity to adenovirus. Hum Gene Ther 25:265–284. doi:10.1089/hum.2014.001. 24512150 PMC3996939

[43] Decker JM, Bibollet-Ruche F, Wei X, Wang S, Levy DN, Wang W, Delaporte E, Peeters M, Derdeyn CA, Allen S, Hunter E, Saag MS, Hoxie JA, Hahn BH, Kwong PD, Robinson JE, Shaw GM. 2005. Antigenic conservation and immunogenicity of the HIV coreceptor binding site. J Exp Med 201:1407–1419. doi:10.1084/jem.20042510. 15867093 PMC2213183

[44] Coutelier JP, van der Logt JT, Heessen FW, Vink A, van Snick J. 1988. Virally induced modulation of murine IgG antibody subclasses. J Exp Med 168:2373–2378. doi:10.1084/jem.168.6.2373. 3199074 PMC2189165

[45] Blutt SE, Miller AD, Salmon SL, Metzger DW, Conner ME. 2012. IgA is important for clearance and critical for protection from rotavirus infection. Mucosal Immunol 5:712–719. doi:10.1038/mi.2012.51. 22739233 PMC3461240

[46] Jogler C, Hoffmann D, Theegarten D, Grunwald T, Uberla K, Wildner O. 2006. Replication properties of human adenovirus in vivo and in cultures of primary cells from different animal species. J Virol 80:3549–3558. doi:10.1128/JVI.80.7.3549-3558.2006. 16537623 PMC1440393

[47] Haynes BF, Gilbert PB, McElrath MJ, Zolla-Pazner S, Tomaras GD, Alam SM, Evans DT, Montefiori DC, Karnasuta C, Sutthent R, Liao HX, DeVico AL, Lewis GK, Williams C, Pinter A, Fong Y, Janes H, DeCamp A, Huang Y, Rao M, Billings E, Karasavvas N, Robb ML, Ngauy V, de Souza MS, Paris R, Ferrari G, Bailer RT, Soderberg KA, Andrews C, Berman PW, Frahm N, De Rosa SC, Alpert MD, Yates NL, Shen X, Koup RA, Pitisuttithum P, Kaewkungwal J, Nitayaphan S, Rerks-Ngarm S, Michael NL, Kim JH. 2012. Immune-correlates analysis of an HIV-1 vaccine efficacy trial. N Engl J Med 366:1275–1286. doi:10.1056/NEJMoa1113425. 22475592 PMC3371689

[48] Rerks-Ngarm S, Pitisuttithum P, Nitayaphan S, Kaewkungwal J, Chiu J, Paris R, Premsri N, Namwat C, de Souza M, Adams E, Benenson M, Gurunathan S, Tartaglia J, McNeil JG, Francis DP, Stablein D, Birx DL, Chunsuttiwat S, Khamboonruang C, Thongcharoen P, Robb ML, Michael NL, Kunasol P, Kim JH, MOPH-TAVEG Investigators. 2009. Vaccination with ALVAC and AIDSVAX to prevent HIV-1 infection in Thailand. N Engl J Med 361:2209–2220. doi:10.1056/NEJMoa0908492. 19843557

[49] Forthal DN, Landucci G, Daar ES. 2001. Antibody from patients with acute human immunodeficiency virus (HIV) infection inhibits primary strains of HIV type 1 in the presence of natural-killer effector cells. J Virol 75:6953–6961. doi:10.1128/JVI.75.15.6953-6961.2001. 11435575 PMC114423

[50] Mielke D, Bandawe G, Pollara J, Abrahams MR, Nyanhete T, Moore PL, Thebus R, Yates NL, Kappes JC, Ochsenbauer C, Garrett N, Abdool Karim S, Tomaras GD, Montefiori D, Morris L, Ferrari G, Williamson C. 2019. Antibody-dependent cellular cytotoxicity (ADCC)-mediating antibodies constrain neutralizing antibody escape pathway. Front Immunol 10:2875. doi:10.3389/fimmu.2019.02875. 31921139 PMC6919271

[51] Román VRG, Murray JC, Weiner LM. 2014. Antibody-dependent cellular cytotoxicity (ADCC), p 1–27. *In* Ackerman ME, Nimmerjahn F (ed), Antibody Fc: linking adaptive and innate immunity. Academic Press, London, United Kingdom. 10.1016/B978-0-12-394802-1.00001-7.

[52] Rahman MA, Ko EJ, Enyindah-Asonye G, Helmold Hait S, Hogge C, Hunegnaw R, Venzon DJ, Hoang T, Robert-Guroff M. 2019. Differential effect of mucosal NKp44(+) innate lymphoid cells and deltagamma cells on simian immunodeficiency virus infection outcome in rhesus macaques. J Immunol 203:2459–2471. doi:10.4049/jimmunol.1900572. 31554692 PMC6810926

[53] Boesch AW, Osei-Owusu NY, Crowley AR, Chu TH, Chan YN, Weiner JA, Bharadwaj P, Hards R, Adamo ME, Gerber SA, Cocklin SL, Schmitz JE, Miles AR, Eckman JW, Belli AJ, Reimann KA, Ackerman ME. 2016. Biophysical and functional characterization of rhesus macaque IgG subclasses. Front Immunol 7:589. doi:10.3389/fimmu.2016.00589. 28018355 PMC5153528

[54] Gao GP, Yang Y, Wilson JM. 1996. Biology of adenovirus vectors with E1 and E4 deletions for liver-directed gene therapy. J Virol 70:8934–8943. doi:10.1128/JVI.70.12.8934-8943.1996. 8971023 PMC190991

[55] Deal C, Pekosz A, Ketner G. 2013. Prospects for oral replicating adenovirus-vectored vaccines. Vaccine 31:3236–3243. doi:10.1016/j.vaccine.2013.05.016. 23707160 PMC3750733

[56] Stephenson KE, Keefer MC, Bunce CA, Frances D, Abbink P, Maxfield LF, Neubauer GH, Nkolola J, Peter L, Lane C, Park H, Verlinde C, Lombardo A, Yallop C, Havenga M, Fast P, Treanor J, Barouch DH. 2018. First-in-human randomized controlled trial of an oral, replicating adenovirus 26 vector vaccine for HIV-1. PLoS One 13:e0205139. doi:10.1371/journal.pone.0205139. 30427829 PMC6235250

[57] Parks CL, Picker LJ, King CR. 2013. Development of replication-competent viral vectors for HIV vaccine delivery. Curr Opin HIV AIDS 8:402–411. doi:10.1097/COH.0b013e328363d389. 23925000 PMC4040527

[58] Patterson LJ, Robert-Guroff M. 2008. Replicating adenovirus vector prime/protein boost strategies for HIV vaccine development. Expert Opin Biol Ther 8:1347–1363. doi:10.1517/14712598.8.9.1347. 18694354 PMC2538611

[59] Peng B, Wang LR, Gomez-Roman VR, Davis-Warren A, Montefiori DC, Kalyanaraman VS, Venzon D, Zhao J, Kan E, Rowell TJ, Murthy KK, Srivastava I, Barnett SW, Robert-Guroff M. 2005. Replicating rather than nonreplicating adenovirus-human immunodeficiency virus recombinant vaccines are better at eliciting potent cellular immunity and priming high-titer antibodies. J Virol 79:10200–10209. doi:10.1128/JVI.79.16.10200-10209.2005. 16051813 PMC1182659

[60] Moore M, Horikoshi N, Shenk T. 1996. Oncogenic potential of the adenovirus E4orf6 protein. Proc Natl Acad Sci U S A 93:11295–11301. doi:10.1073/pnas.93.21.11295. 8876129 PMC38051

[61] Freeman ML, Shive CL, Nguyen TP, Younes SA, Panigrahi S, Lederman MM. 2016. Cytokines and T-cell homeostasis in HIV infection. J Infect Dis 214(Suppl 2):S51–S57. doi:10.1093/infdis/jiw287. 27625431 PMC6373575

[62] Seif F, Khoshmirsafa M, Aazami H, Mohsenzadegan M, Sedighi G, Bahar M. 2017. The role of JAK-STAT signaling pathway and its regulators in the fate of T helper cells. Cell Commun Signal 15:23. doi:10.1186/s12964-017-0177-y. 28637459 PMC5480189

[63] Murakami M, Kamimura D, Hirano T. 2019. Pleiotropy and specificity: insights from the interleukin 6 family of cytokines. Immunity 50:812–831. doi:10.1016/j.immuni.2019.03.027. 30995501

[64] Floss DM, Moll JM, Scheller J. 2020. IL-12 and IL-23—close relatives with structural homologies but distinct immunological functions. Cells 9:2184. doi:10.3390/cells9102184. PMC7600943 32998371

[65] Hasegawa H, Mizoguchi I, Chiba Y, Ohashi M, Xu M, Yoshimoto T. 2016. Expanding diversity in molecular structures and functions of the IL-6/IL-12 heterodimeric cytokine family. Front Immunol 7:479. doi:10.3389/fimmu.2016.00479. 27867385 PMC5095122

[66] Floss DM, Schonberg M, Franke M, Horstmeier FC, Engelowski E, Schneider A, Rosenfeldt EM, Scheller J. 2017. IL-6/IL-12 cytokine receptor shuffling of extra- and intracellular domains reveals canonical STAT activation via synthetic IL-35 and IL-39 signaling. Sci Rep 7:15172. doi:10.1038/s41598-017-15173-3. 29123149 PMC5680241

[67] Forthal DN, Landucci G, Cole KS, Marthas M, Becerra JC, Van Rompay K. 2006. Rhesus macaque polyclonal and monoclonal antibodies inhibit simian immunodeficiency virus in the presence of human or autologous rhesus effector cells. J Virol 80:9217–9225. doi:10.1128/JVI.02746-05. 16940533 PMC1563916

[68] Ackerman ME, Dugast AS, McAndrew EG, Tsoukas S, Licht AF, Irvine DJ, Alter G. 2013. Enhanced phagocytic activity of HIV-specific antibodies correlates with natural production of immunoglobulins with skewed affinity for FcgammaR2a and FcgammaR2b. J Virol 87:5468–5476. doi:10.1128/JVI.03403-12. 23468489 PMC3648186

[69] Watkins JD, Sholukh AM, Mukhtar MM, Siddappa NB, Lakhashe SK, Kim M, Reinherz EL, Gupta S, Forthal DN, Sattentau QJ, Villinger F, Corti D, Ruprecht RM, CAVD Project Group. 2013. Anti-HIV IgA isotypes: differential virion capture and inhibition of transcytosis are linked to prevention of mucosal R5 SHIV transmission. AIDS 27:F13–F20. doi:10.1097/QAD.0b013e328360eac6. 23775002 PMC4084966

[70] National Research Council. 2011. Guide for the care and use of laboratory animals, 8th ed. National Academies Press, Washington, DC.

[71] Barker DD, Berk AJ. 1987. Adenovirus proteins from both E1B reading frames are required for transformation of rodent cells by viral infection and DNA transfection. Virology 156:107–121. doi:10.1016/0042-6822(87)90441-7. 2949421

[72] Patterson LJ, Daltabuit-Test M, Xiao P, Zhao J, Hu W, Wille-Reece U, Brocca-Cofano E, Kalyanaraman VS, Kalisz I, Whitney S, Lee EM, Pal R, Montefiori DC, Dandekar S, Seder R, Roederer M, Wiseman RW, Hirsch V, Robert-Guroff M. 2011. Rapid SIV Env-specific mucosal and serum antibody induction augments cellular immunity in protecting immunized, elite-controller macaques against high dose heterologous SIV challenge. Virology 411:87–102. doi:10.1016/j.virol.2010.12.033. 21237474 PMC3039060

[73] Xiao P, Patterson LJ, Kuate S, Brocca-Cofano E, Thomas MA, Venzon D, Zhao J, DiPasquale J, Fenizia C, Lee EM, Kalisz I, Kalyanaraman VS, Pal R, Montefiori D, Keele BF, Robert-Guroff M. 2012. Replicating adenovirus-simian immunodeficiency virus (SIV) recombinant priming and envelope protein boosting elicits localized, mucosal IgA immunity in rhesus macaques correlated with delayed acquisition following a repeated low-dose rectal SIV_mac251_ challenge. J Virol 86:4644–4657. doi:10.1128/JVI.06812-11. 22345466 PMC3318604

[74] Montefiori DC. 2005. Evaluating neutralizing antibodies against HIV, SIV, and SHIV in luciferase reporter gene assays. Curr Protoc Immunol Chapter12:Unit 12.11. doi:10.1002/0471142735.im1211s64. 18432938

